# Psychometric properties of the full‐form and short‐form versions of the Integration of Stressful Life Experiences Scale (ISLES) among Iranian university students

**DOI:** 10.1002/brb3.2892

**Published:** 2023-02-14

**Authors:** Zahra Azadfar, Azam Farah Bijari, Zohreh Khosravi, Abbas Abdollahi

**Affiliations:** ^1^ Department of Psychology, Faculty of Education and Psychology Alzahra University Tehran Iran; ^2^ Department of Counseling, Faculty of Education and Psychology Alzahra University Tehran Iran

**Keywords:** Iranian, meaning‐made, psychometric, romantic breakup, university students

## Abstract

**Background:**

Making adaptive meaning of stressful life experiences has been identified as an important determinant of adjustment. The Integration of Stressful Life Experiences Scale (ISLES) was developed to assess the outcome of meaning‐making processes in the face of negative events.

**Aims:**

The psychometric properties of this scale have not been measured in Iranian populations. The purpose of the present study was to examine the psychometric properties of the 16‐item and 6‐item versions of ISLES with a sample of 502 university students who had experienced relationship dissolution.

**Results:**

Findings support a two‐factor structure with acceptable validity and reliability. Positive correlations between the scores of ISLES with Centrality of Event Scale and PTSD symptoms provided evidence of concurrent validity.

**Conclusion:**

The results of measurement invariance indicated that both the 16‐item and 6‐item versions of ISLES are gender invariant and can be used to assess meaning‐made in both men and women.

## Graphical abstract

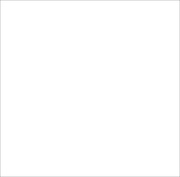

The effort to create and maintain a sense of meaning, coherence, and purpose in life is central to human existence. Meaning seems to be particularly important when confronting stressful life events in which people may experience existential challenges (Park, 2010; Park & Folkman, 1997). Park (2010) in an integrative model of meaning‐making has distinguished between g*lobal meaning* and *situational meaning*. Global meaning involves global beliefs and goals that contribute in the sense of purpose and meaningfulness in life and assist in interpreting life experiences. Situational meaning refers to the appraisals of a stressful life event. The cognitive appraisal of an event as threatening and uncontrollable can violate one's belief systems about the meaningfulness of life and the predictability of the world (Park & George, 2013). For example, a survivor of violence may evaluate the event in a way that challenges his/her core assumptions about the benevolence of people and the safety of the world.

The central component of Park's (2010) model is that the perceived discrepancy between the situational appraised meaning and the global meaning system causes distress that initiates the meaning‐making process. In the meaning‐making process, individuals attempt to create coherence between the global meaning and the situational meaning through *assimilation* (changing the appraised meaning of the event to fit one's worldviews) or *accommodation* (changing one's global goals or beliefs to match the situational meaning) to reduce the experienced distress. Meaning‐making includes efforts to understand the meaning of a specific event and the search for its significance. Comprehension of an event is often identified as an assimilative process, whereas finding a sense of significance in a stressful experience is more an accommodative process (Janoff‐Bulman & McPherson Frantz, 1997; Joseph & Linley, 2005; Park, 2010).

Meaning‐making is often described as a critical aspect of adjustment after experiencing stressful life events. However, meaning‐making does not necessarily lead to better adjustment. Prolonged search for meaning without any outcome usually leads to increased distress and rumination. Meaning‐made is the ultimate product of successful meaning‐making or adaptive integration of an event into one's life narrative. Meaning‐made often consists of acceptance, perceived growth, reattributions, altered identity, viewing the event as less aversive or threatening, changed global goals or beliefs, and recovered sense of meaning in life (Park, 2010; Park & George, 2013). The only measure that assesses meaning‐made or the product of the dual processes (assimilation/accommodation and comprehensibility/significance) of meaning‐making according to Park's (2010) model is the Integration of Stressful Life Experiences Scale (ISLES) (Holland et al., 2010). The ISLES is a self‐reported multidimensional measure developed to assess meaning‐made that includes two factors: *Footing in the World* represents the extent to which an individual's global worldviews have been altered to accommodate the stressful experience. *Comprehensibility* measures the extent to which an individual was able to make sense of an event or assimilate it into one's existing meaning structures (Holland et al., 2010; Holland, Currier, et al., 2014).

Some measures have been validated in Iran to assess meaning‐related constructs. The Sense of Coherence Scale (Alipour & Sharif, 2012) measures one's perception of the comprehensibility, manageability, and meaningfulness of life, which promotes coping with stress and engaging in health‐related behaviors. This measure captures the global sense of orientation and coherence in life and does not reflect the subjective sense of meaning or sense‐making in dealing with a particular stressor (Park & George, 2013). The Meaning in Life Questionnaire (MLQ; Mesrabadi et al., 2013) measures the presence of meaning and the search for meaning in life. The MLQ measures the general sense of meaning in life, whereas examining the sense‐making after a stressful life event requires a more event‐oriented scale (Lancaster & Carlson, 2015). Recently, the Posttraumatic Growth Inventory (PTGI; Mahmoudi et al., 2013) has been widely used in Iran to assess individuals’ experience of positive changes in various life domains as a result of a stressful life event. The PTGI is assumed to comprise two constructive and self‐deceptive aspects and has shown inconsistent associations with distress and adjustment (Zoellner & Maercker, 2006). Research suggests that meaning‐made is an adaptive product associated with active coping, whereas posttraumatic growth is associated with both positive growth and ongoing distress (Park et al., 2012). Therefore, the ISLES is unique in that it assesses the degree to which a stressful experience has been adaptively integrated into one's life story that promotes better adjustment (Holland et al., 2010).

Holland et al. (2010) developed the English version of ISLES and examined its psychometric properties in 150 bereaved adults and 178 adults with a range of experienced stressful life events. Results from the exploratory factor analysis supported a two‐factor structure according to the theoretical model. Excellent internal consistency was found for the Footing in the World factor and total ISLES (0.92–0.94 in two studied samples). In addition, the internal consistency coefficient for the comprehensibility factor was 0.80 and 0.85 for the bereaved and general stress samples, respectively. Test–retest reliability was moderate (*r* = .48 to *r* = .59) over 3 months. In addition, the results of follow‐up analyses indicated that the scores of ISLES over time appeared to show significant changes in psychological distress and complicated grief symptoms. Convergent validity (CV) results indicated that the two factors of the ISLES were negatively correlated with depression, PTSD, and grief symptoms and positively correlated with perceived general health. Lancaster and Carlson (2015) examined the convergent and incremental validity of the ISLES in 234 undergraduates with a variety of stressful life experiences. Results indicated that the two factors of ISLES were uniquely correlated with PTSD and depressive symptoms, over and above the other meaning‐related measures. Currier et al. (2013) examined the psychometric properties of the Spanish version of the ISLES. Results from the confirmatory factor analysis (CFA) supported a two‐factor structure. In addition, Thimm and Holland (2017) reported Cronbach's alpha of .92 for a Norwegian version.

Because the ISLES has shown good psychometric properties in related studies, Holland, Rengifo et al. (2014) developed a brief 6‐item version among 741 bereaved adults who had lost a loved one in the past 2 years. The ISLES‐Short Form (ISLES‐SF) yielded a two‐factor structure like the original version. Footing in the World and comprehensibility were constructed from the three items with the highest factor loadings on each of the two factors. The ISLES‐SF correlated highly with the original ISLES for Footing in the World (*r* = .92, *p* < .001), comprehensibility (*r* = .93, *p* < .001), and the total ISLES (*r* = .95, *p* < .001). The ISLES‐SF showed similar convergent and incremental validity in relation to positive and negative psychological outcomes (Holland, Currier, et al., 2014). Milman et al. (2020) reported Cronbach's alpha of .86 for Footing in the World, 0.78 for comprehensibility, and 0.87 for the total ISLES‐SF.

Research using the 16‐item and 6‐item versions of the ISLES found that meaning‐made was associated with fewer PTSD symptoms and referral to mental health services among US veterans (Currier et al., 2011), less burnout and psychiatric symptomatology among Salvadorian teachers exposed to violence (Currier et al., 2013), decreased salivary cortisol in depressed older adults (Holland, Rengifo, et al., 2014), less suicidal and life‐threatening behaviors in military veterans (Holland, Malott, et al., 2014), less grief symptomatology in bereaved young adults (Thimm & Holland, 2017), and less anxiety and depression in adults exposed to coronavirus in the United States (Milman et al., 2020).

Validating a measure to assess the meaning‐made of stressful experiences can provide psychometrically sound instruments to examine whether a negative experience has been adaptively incorporated into one's autobiographical knowledge. However, there is currently no measure in Persian to assess the product of meaning‐making processes in the face of adversity. All studies that examined the psychometric properties of ISLES were conducted in Western countries, whereas research has shown that culture and religion strongly influence searching for meaning and making sense of stressful life experiences (Ahmadi et al., 2019; Lewis Hall & Hill, 2019). Hence, it seems necessary to assess the validity and reliability of ISLES in Eastern cultures and highly religious countries, including Iran. Therefore, the present study aimed to measure the psychometric properties of the Persian version of the 16‐item and 6‐item forms of the ISLES among Iranian university students who have experienced at least 1 romantic breakup in the past 2 years. The loss of a romantic partner or attachment figure (either as a result of death or relationship dissolution) is one of the most stressful losses among young adults (Boelen & van den Hout, 2010). Research indicated that individuals may experience posttraumatic stress symptoms following the dissolution of a romantic relationship (Chung & Hunt, 2014; Chung et al., 2002, 2003). Bereavement research has shown that making adaptive meaning of the loss experience is related to less PTSD and grief symptoms (Bellet et al., 2018; Rozalski et al., 2017).

Event centrality is one of the strong cognitive correlates of PTSD that refers to the extent to which a traumatic memory has become a central part of one's identity and life story (Berntsen & Rubin, 2006). Although meaning‐made represents the adaptive integration of an event into one's meaning systems, event centrality exhibits over the integration of an event into one's self‐narratives (Bellet et al., 2018; Currier et al., 2013). Accordingly, we expected that meaning‐made to be negatively correlated with the Centrality of Event Scale (CES; Berntsen & Rubin, 2006). The potential gender differences in making sense of stressful or traumatic life events have not been clarified in Park's (2010) model of meaning‐making. Research on meaning‐making processes has also yielded conflicting results regarding gender differences in meaning‐made following traumatic/stressful life experiences. Some studies suggest that gender may influence the relationship between meaning‐made and psychological outcomes (Holland, Currier, et al., 2014; Lancaster & Carlson, 2015). Holland, Currier et al. (2014) showed that women tended to score significantly lower on the comprehensibility subscale of ISLES. However, Currier et al. (2013) reported that men are less likely to make sense of traumatic/stressful life experiences than women. On the other hand, Currier et al. (2011) found no gender differences in the meaning‐made after traumatic/stressful life experiences. Therefore, it seems necessary to further investigate gender differences in making sense of traumatic/stressful events between men and women. In order to investigate whether gender differences actually exist in a construct, the gender invariance of the measurement model between males and females must first be ascertained. Accordingly, the measurement invariance of the ISLES was also examined in the current study.

Therefore, the present study aimed to examine the psychometric properties of the Persian version of the ISLES and ISLES‐SF among Iranian university students who experienced a romantic breakup in the past 2 years. We hypothesized that both 16‐item and 6‐item versions of ISLES would have a two‐factor structure and good internal consistency (hypothesis 1), the scores of both 16‐item and 6‐item versions of ISLES would negatively correlate with CES and PTSD symptoms (hypothesis 2), and these two versions of ISLES would be gender invariant (hypothesis 3).

## 1 METHODS

### 1.1 Participants

Participants were 502 university students aged 18–25 who had experienced at least 1 romantic breakup in the past 2 years. The mean age of participants was 21.92 (SD = 2.26), and the majority of them were female (*n* = 382, 76%). A total of 381 (76%) of the students were undergraduates, and 121 (24%) were postgraduates. In terms of initiator status, 253 (50%) participants were the initiator of the breakup. In terms of relationship duration, 147 (29%) participants were in a romantic relationship for less than 6 months, 106 (21%) were in a romantic relationship for 6 months to 1 year, 94 (19%) were in a romantic relationship for 1–2 years, and 155 (31%) were in a romantic relationship for more than 2 years. In addition, 253 (50%) of the participants had experienced a highly committed romantic relationship, 187 (37%) were in a relationship with a medium level of commitment, and 62 (13%) of the participants were in a relationship with a low level of commitment. In terms of breakup distress, 349 (69%) of participants reported high levels of perceived breakup distress, 120 (24%) reported a medium level of distress, and 33 (7%) reported a low level of distress (see Table 1).

**TABLE 1:** Participant demographics

**Variable**	**Percent (*n*)**
**Relationship duration**	
Less than 6 months	29% (*n* = 147)
Between 6 months and 1 year	21% (*n* = 106)
Between 1 and 2 years	19% (*n* = 94)
More than 2 years	31% (*n* = 155)
**Relationship commitment and intimacy**	
Low	12% (*n* = 62)
Moderate	37% (*n* = 187)
High	51% (*n* = 253)
**The initiator status**	
Initiator	50% (*n* = 253)
Non‐initiator	50% (*n* = 249)
**Breakup distress**	
Low	7% (*n* = 33)
Moderate	24% (*n* = 120)
High	69% (*n* = 349)

### 1.2 Instruments

#### 1.2.1 The Integration of Stressful Life Experiences Scale

ISLES (Holland et al., 2010) is a 16‐item self‐reported questionnaire to assess the degree to which an individual has made meaning of a stressful life experience. This measure is scored on a 5‐point Likert scale from 1 (*strongly agree*) to 5 (*strongly disagree*) to evaluate 2 subscales of Footing in the World that consists of 11 items (e.g., “*Since this event, I feel like I'm in a crisis of faith*”) and comprehensibility that includes 5 items (e.g., “*This event is incomprehensible to me*.”). The items of ISLES, except for item 2, are negatively worded, so item 2 should be reverse‐scored. A higher score in each subscale specifies a greater level of adaptive integration of a specific stressor (Holland et al., 2010). In the previous studies, Cronbach's alpha coefficient for the total ISLES was reported in the range of 0.92–0.96 (Currier et al., 2011; Holland et al., 2010; Lichtenthal et al., 2011; Thimm & Holland, 2017).

The translation of the English version of ISLES was carried out according to the method of Brislin (1986). Two expert translators fluent in both English and Persian were independently asked to translate the ISLES. One of the translators was asked to translate the ISLES into Persian. Then the second translator asked to translate the Persian version back into English without knowing the first translation. Finally, three independent translators compared these two versions of ISLES in terms of content and concept and reported that there were no significant differences between the Persian and the original versions.

#### 1.2.2 The Integration of Stressful Life Experiences Scale‐Short Form

ISLES‐SF (Holland, Currier, et al., 2014) is a short 6‐item version of the ISLES comprising 2 factors: Footing in the World (Items 9, 12, and 14) and comprehensibility (Items 4, 6, and 8). This short version derived from the original ISLES and indicated good internal consistency (*a* = .87 to *a* = .96) in the previous studies (Milman et al., 2020; Slagel et al., 2020). To evaluate the psychometric properties of both the 16‐item and 6‐item versions of the ISLES, scores for both these versions were calculated based on the participant's answers to the original version.

#### 1.2.3 The Centrality of Event Scale

CES (Berntsen & Rubin, 2006) is a self‐reported measure to assess the extent to which a traumatic memory has become a central part of an individual's identity and life story (e.g., “*I feel that this event has become a central part of my life story*.”). The items are scored on a 5‐point Likert scale from 1 (*totally disagree*) to 5 (*totally agree*) so that higher scores indicate greater centrality of an event in one's identity and life story. The 7‐item version of this scale has shown good psychometric properties (with alphas between .88 and .93) in related studies (Berntsen & Rubin, 2006; Vermeulen et al., 2020). Recently, the Persian version of the 7‐item CES has been validated in Iran and indicated good psychometric properties with Cronbach's alpha of .86 (Azadfar et al., 2022).

#### 1.2.4 The Posttraumatic Stress Disorder Checklist for DSM5

The Posttraumatic Stress Disorder Checklist for DSM5 (Blevins et al., 2015; Weathers et al., 2013) is a 20‐item self‐reported measure to evaluate the severity of posttraumatic stress disorder symptoms, including reexperiencing (e.g., “*Repeated, disturbing dreams of the stressful experience?*.”), avoidance (e.g., “*Avoiding memories, thoughts, or feelings related to the stressful experience?*”), negative alterations in cognitions and mood (e.g., “*Trouble remembering important parts of the stressful experience?*”), and hyperarousal (e.g., “*Irritable behavior, angry outbursts, or acting aggressively?*”). This measure uses a 5‐point Likert scale from 0 (*Not at all*) to 4 (*Extremely*) that a higher score represents higher severity of PTSD symptoms (Blevins et al., 2015; Weathers et al., 2013). The Persian version of this scale has shown good internal consistency with Cronbach's alpha of .67–.90 (Varmaghani et al., 2018).

### 1.3 Procedure

Participants were invited to take part in a study about dealing with a romantic breakup via an advertisement shared on the social networks of several universities in Tehran. The advertisement included a brief description of the research aims (to examine the psychological effects of a romantic breakup). The inclusion criteria consisted of (1) college students between the ages of 18 and 25, (2) who had experienced at least one romantic breakup within the past 2 years, and (3) who were willing to participate in the present study. Students who met the inclusion criteria participated in the online survey via a hyperlink on the Porsa online survey platform. Before completing the research questionnaires, participants were shown a page explaining the ethical considerations of the research. Participants provided some information about their most stressful romantic breakup and then answered the main questionnaires. Participants were also asked to complete the questionnaires in reference to their romantic breakup. Data collection was conducted for 1 month, from April to May 2021. The approximate time to answer the questions was about 20 min. The procedure and research materials of the present study were reviewed and approved by the Ethics Committee at Alzahra University. Participants were informed that participation in this study was voluntary and that they had the right to withdraw from the study at any time. They were also assured that all their information would be kept confidential and anonymous.

### 1.4 Data analysis

Preliminary data screening was performed using the Asset Management Operating System (AMOS 24). As responses to each item were required for submission of the online questionnaires, there were no missing data. Outliers were assessed using the Mahalanobis distance in the AMOS software. Dividing the maximum Mahalanobis distance value (38.46) by the number of indicators (16) resulted in a value of 2.4. Because the resulting value was less than 4, there were no outliers in the data set (Tabachnick & Fidell, 2014). The normal distribution of the data was determined using the skewness and kurtosis values in the AMOS software. The results of the normality analysis showed that the skewness (−0.58 to 0.54) and kurtosis (−0.14 to −1.25) values were within the ranges of ±2 and ±3, respectively, so the data set had an acceptable normal distribution (Tabachnick & Fidell, 2014).

To evaluate the qualitative face validity of the Persian version of the ISLES, we selected eight university student volunteers as potential participants who did not take part in the main study and instructed them to review the questions of the measure and describe the relevance, difficulty, and ambiguity of each item. It is recommended to use at least five people for face and content validation to have sufficient control over chance agreement (Yusoff, 2019; Zamanzadeh et al., 2015). After the corrections (in the wording of some questions to make them more understandable for the participants) based on their comments, the same eight students were asked to answer a questionnaire to assess the quantitative face validity of the ISLES. For this purpose, they were instructed to rate the relevance, appropriateness, difficulty, and ambiguity of each item on a 5‐point Likert scale ranging from 1 (*not important*) to 5 (*completely important*). Quantitative face validity was calculated using the impact score index. The formula for calculating the impact score is to multiply the percentage frequency of participants who selected a value of 4 or 5 for the item by the mean value of that item. An impact score value of 1.5 or more is an index of acceptable face validity (Birring et al., 2003; Hajizadeh & Asghari, 2010).

The qualitative content validity of the ISLES was confirmed by the opinions of eight experts (clinical psychologists with Ph.D. degree and at least 5 years of experience who were familiar with meaning‐making processes, selected by the authors via convenience sampling) on the relevance and appropriateness of each statement in the questionnaire. Slight modifications (in the wording of some questions) were made based on the experts’ comments. Then, the quantitative content validity of the ISLES was assessed using the content validity index (CVI) and the content validity ratio (CVR) (Cook & Beckman, 2006). The CVR assesses the essentiality of each item, which was to be rated by the same eight experts on a 3‐point Likert scale ranging from (1) *not essential* to (3) *essential*. A CVR score greater than 0.75 indicates acceptable content validity for that item (LAWSHE, 1975). The CVI assesses the simplicity, clarity, and relevance of indicators based on experts’ opinions. The experts were asked to rate their opinions on each item on a 4‐point Likert scale ranging from (1) *not relevant at all* to (4) *very relevant*. A CVI value greater than 0.7 for each item indicates satisfactory content validity (Cook & Beckman, 2006).

A CFA with maximum likelihood estimation in the AMOS software was applied to assess the factor structure of the two 6‐item and 16‐item versions of the ISLES. According to Kellar and Kelvin (2013), acceptable sample size for conducting CFA is equal to 5–20 times more than the sum of the items in the measure. Considering the potential outliers, 502 university students were recruited to participate in this study. The concurrent validity of the 16‐item and 6‐item versions of ISLES was assessed based on the relationship between ISLES and ISLES‐SF scores and related constructs.

Testing for measurement invariance determines whether a construct has the same meaning across different groups. Measurement invariance is an essential component of psychological research and a prerequisite for mean comparisons across groups. Therefore, an applicable comparison of a psychological construct between different groups depends first on establishing the construct's equality of meaning across target groups (Putnick & Bornstein, 2016). To test the measurement invariance of the ISLES and ISLES‐SF within gender groups, we first randomly selected 120 females using SPSS (because the sample size was unbalanced between males [*n* = 120] and females [*n* = 382]). According to the guidelines provided by Vandenberg and Lance (2000), measurement invariance was tested in increasingly restrictive stages. In the first stage, the test for *configural invariance* (baseline model) was conducted to examine the equality of total factor structure across gender groups. In the second step, *metric invariance* model was tested, in which the factor loadings of the items were constrained to be the same in the two groups. Then, the metric invariance model was compared with the configural invariance model. In the third step, the *scalar invariance* model was tested, in which the factor loadings and item intercepts were constrained to be the same in the two groups. Testing for scalar invariance is required before we can compare latent means between groups. Metric and scalar invariances were determined by assessing the changes in *χ* ^2^ and comparative fit index (CFI) in the model (Cheung & Rensvold, 2002).

The full scalar invariance model was employed as the baseline for comparing latent mean differences between men and women. Using the reference group method (Tabri & Elliott, 2012), the means for the latent constructs in the female group were constrained to be 0. The latent mean values of the constructs in the male group were freely estimated and showed latent mean differences between these two groups. The value of the critical ratio (CR) was used to evaluate latent mean differences between males and females. A CR value greater than 1.96 represents significant latent mean differences across groups.

## 2 RESULTS

### 2.1 Face validity

According to the quantitative face validity results, the impact score values of all indicators were greater than 1.5, which supports the acceptable face validity of the ISLES.

### 2.2 Content validity

The CVR values for all indicators were greater than the Lawshe value (0.75), indicating that all items had sufficient content validity (Lawshe, 1975). The results also showed that the CVI scores for all indicators were greater than 0.7, indicating satisfactory content validity for all the items (see Table 2).

**TABLE 2:** Content validity ratio (CVR) and content validity index (CVI) for the items of Integration of Stressful Life Experiences Scale (ISLES)

		CVI			CVR
No	Items	Simplicity (1–4)	Relevancy (1–4)	Clarity (1–4)	Essential (1–3)
1	Since this event, the world seems like a confusing and scary place	1	1	1	1
2	I have made sense of this event	1	1	0.87	1
3	If or when I talk about this event, I believe people see me differently	1	1	0.87	1
4	I have difficulty integrating this event into my understanding about the world	0.87	1	1	1
5	Since this event, I feel like I'm in a crisis of faith	1	1	1	1
6	This event is incomprehensible to me	1	1	1	1
7	My previous goals and hopes for the future don't make sense anymore since this event	1	1	1	1
8	I am perplexed by what happened	1	1	1	1
9	Since this event happened, I don't know where to go next in my life	1	1	1	1
10	I would have an easier time talking about my life if I left this event out	1	0.87	1	1
11	My beliefs and values are less clear since this event	1	1	1	1
12	I don't understand myself anymore since this event	0.87	0.87	1	0.75
13	Since this event, I have a harder time feeling like I'm part of something larger than myself	0.87	1	0.75	0.75
14	This event has made me feel less purposeful	1	1	1	1
15	I haven't been able to put the pieces of my life back together since this event	1	1	1	1
16	After this event, life seems more random	1	1	1	1

*Note*. CVR for an item was calculated using the formula: $R=\frac{ne-N/2}{n/2}$. The *ne* here is the number of experts who selected the score of 3 for a particular item, and *N* is the number of experts. The following formula was used to calculate the CVI for an item: $\mathrm{CVI}=\frac{ne}{N}$. The *ne* here in the number of experts who selected the score of 3 or 4 for an item, and *N* is the total number of experts.

### 2.3 Construct validity

**FIGURE 1:**
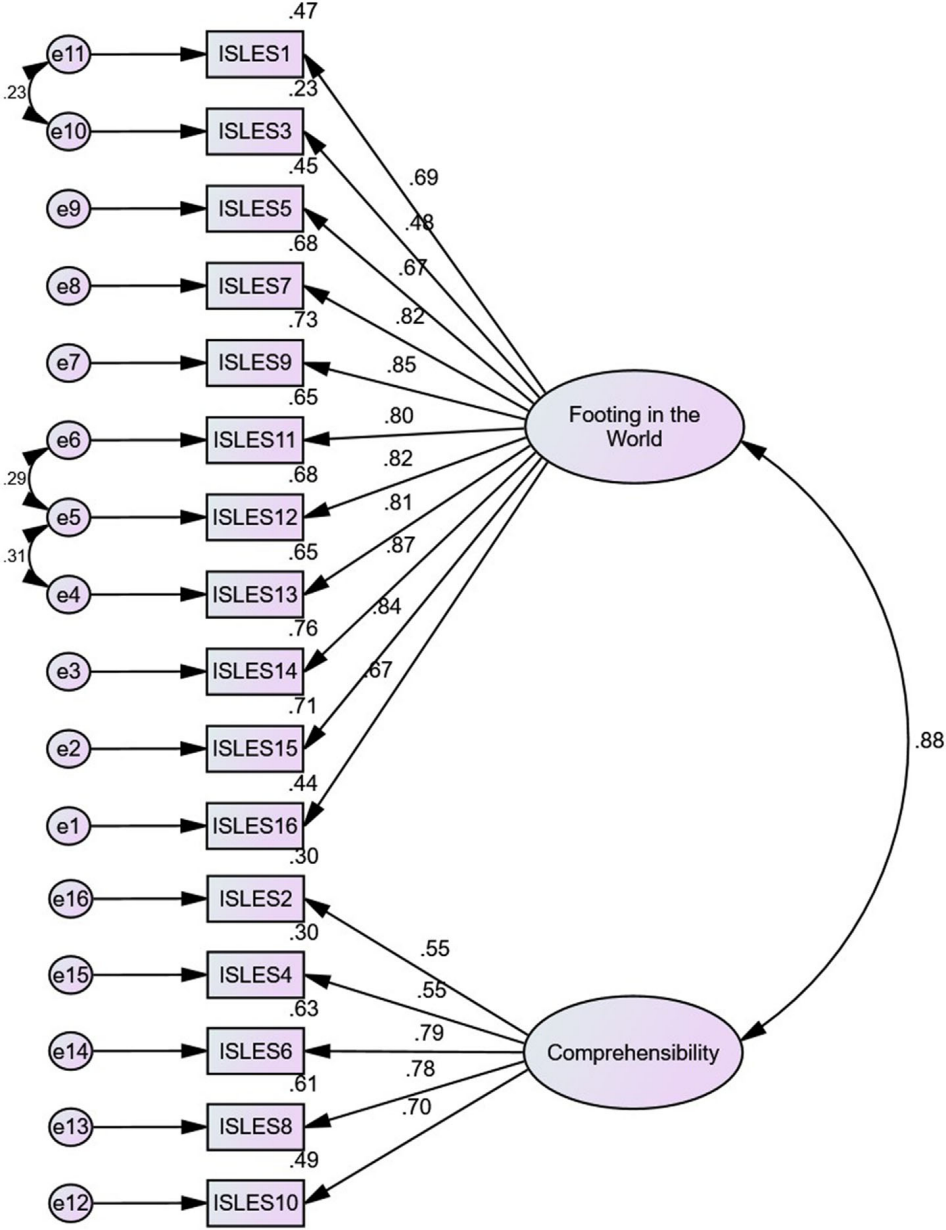
Confirmatory factor analysis with factor loadings for the two subscales of Integration of Stressful Life Experiences Scale (ISLES) (*p* < 001).

**FIGURE 2:**
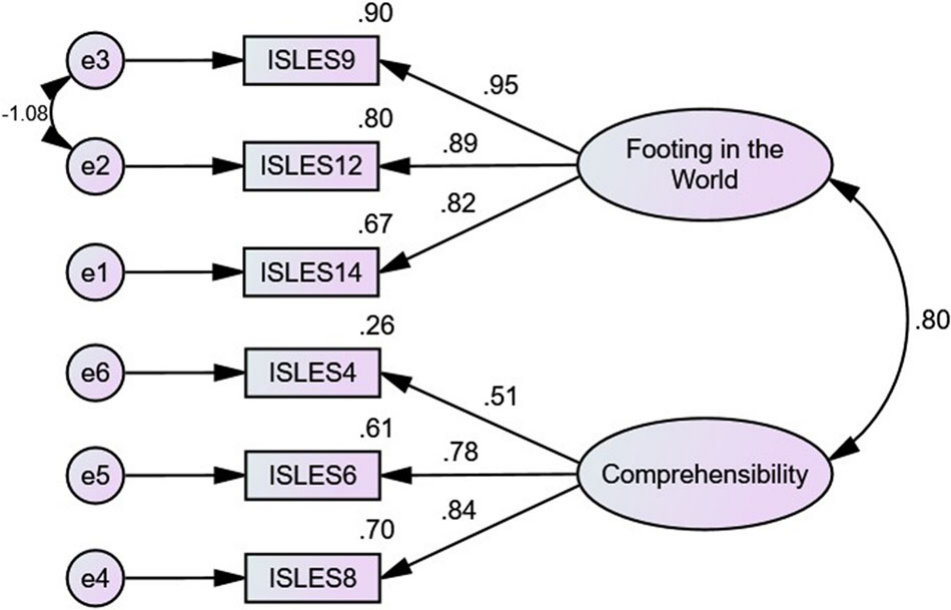
Confirmatory factor analysis with factor loadings for the two subscales of Integration of Stressful Life Experiences Scale‐Short Form (ISLES‐SF) (*p* < 001).

The factor loadings of all indicators ranged from 0.48 to 0.87 in ISLES (see Figure 1) and 0.51 to 0.95 in ISLES‐SF (see Figure 2). The results of CFA showed that the factor loadings of all items were greater than 0.4, less than 1, and none of them were negative (Tabri & Elliott, 2012); therefore, all indicators of ISLES and ISLES‐SF were left in the measure. The means and standard deviations of all indicators are shown in Table 3.

**TABLE 3:** Means and standard deviations of the items of the Integration of Stressful Life Experiences Scale (ISLES)

No	Items	Mean	Std. deviation
1	Since this event, the world seems like a confusing and scary place	3.18	1.36
2	I have made sense of this event	3.55	1.01
3	If or when I talk about this event, I believe people see me differently	3.07	1.18
4	I have difficulty integrating this event into my understanding about the world	2.74	1.15
5	Since this event, I feel like I'm in a crisis of faith	3.25	1.38
6	This event is incomprehensible to me	3.28	1.28
7	My previous goals and hopes for the future don't make sense anymore since this event	3.57	1.36
8	I am perplexed by what happened	3.08	1.33
9	Since this event happened, I don't know where to go next in my life	3.52	1.39
10	I would have an easier time talking about my life if I left this event out	2.82	1.28
11	My beliefs and values are less clear since this event	3.28	1.32
12	I don't understand myself anymore since this event	3.49	1.29
13	Since this event, I have a harder time feeling like I'm part of something larger than myself	3.49	1.25
14	This event has made me feel less purposeful	3.52	1.35
15	I haven't been able to put the pieces of my life back together since this event	3.53	1.22
16	After this event, life seems more random	2.53	1.27

The following fit indices were used to test the measurement model of the ISLES and ISLES‐SF: Normed chi‐squared (CMIN/df), CFI, incremental fit index (IFI), root mean squared error of approximation (RMSEA), and standardized root mean square residual (SRMR). The model fit is considered acceptable when the value of CMIN/df is less than 5, CFI and IFI are above .9, RMSEA is less than .10, and SRMR is less than .09 (Hu & Bentler, 1998). The results showed that the 16‐item ISLES with a two‐factor structure adequately fit the data (CMIN/df = 4.94, *p* < .01, CFI = .93, IFI = .93, RMSEA = .089, and SRMR = .047). CFA results also supported a good model fit of the 6‐item ISLES with two subscales (CMIN/df = 2.13, *p* < .01, CFI = .99, IFI = .99, RMSEA = .048, and SRMR = .014).

The CV of the two factors of the ISLES and ISLES‐SF was tested using the average variance extracted (AVE). An AVE greater than 0.5 indicates acceptable CV. Although the AVE value of comprehensibility of the ISLES was less than the threshold of 0.5 (0.47), as the CR value of this construct was greater than 0.6, the CV value is also acceptable (Fornell & Larcker, 1981). Therefore, the items of each subscale had satisfactory CV for the associated construct (see Table 4). The assessment of internal consistency and reliability of the two factors of the ISLES and ISLES‐SF was conducted using the composite reliability (CR) value and Cronbach's alpha coefficient (*a*). The results showed that the CR and Cronbach's alpha values were greater than .7, suggesting that the two factors of the ISLES and ISLES‐SF have satisfactory construct reliability (see Table 4).

**TABLE 4:** Average variance extracted (AVE), CR, and Cronbach's alpha for two subscales of Integration of Stressful Life Experiences Scale (ISLES) and Integration of Stressful Life Experiences Scale‐Short Form (ISLES‐SF)

Variables	AVE	CR	Cronbach's alpha
**ISLES**			
Footing in the World	.59	.94	.94
Comprehensibility	.47	.81	.80
**ISLES‐SF**			
Footing in the World	.79	.92	.89
Comprehensibility	.52	.76	.75

### 2.4 Concurrent validity

Correlations between the scores of ISLES and ISLES‐SF with measures of event centrality and PTSD symptoms are presented in Table 6. Based on Cohen's (1992) guidelines for large, medium, and small effect sizes, two factors of ISLES and ISLES‐SF showed moderate negative correlations with event centrality (*r* = −.47 and −.45, *p* < .01, ISLES and ISLES‐SF, respectively) and large negative correlations with overall PTSD symptomatology (*r* = −.68 and −.66, *p* < .01, ISLES and ISLES‐SF, respectively).

### 2.5 Measurement invariance across gender

The results of the multigroup analyses in the AMOS software confirmed that the configural invariance model indicated acceptable model fit across gender groups in both the 16‐item and 6‐item versions of ISLES (see Table 5). As shown in Table 5, the results of the chi‐square difference test indicated that constraining factor loadings (metric invariance) and intercepts (scalar invariance) did not significantly change the model fit. Moreover, the change from CFI was smaller than the threshold of 0.01. Therefore, both the 16‐item and 6‐item versions of ISLES are gender invariant, allowing for mean comparisons between gender groups Table 6.

**TABLE 5:** Measurement invariance tests of Integration of Stressful Life Experiences Scale (ISLES) and Integration of Stressful Life Experiences Scale‐Short Form (ISLES‐SF) across genders

Model	*χ* ^2^	df	CFI	RMSEA (90%CI)	SRMR	Δ*χ* ^2^ *	Δdf^a^	Sig.	ΔCFI^b^
**ISLES**									
Configural invariance	434.543	200	.907	.070 (.061–.079)	.060				
Full metric invariance	444.261	214	.909	.067 (.058–.076)	.064	9.718	14	.782	.002
Full scalar invariance	462.829	228	.907	.066 (.057–.074)	.064	18.568	14	.182	.002
**ISLES‐SF**									
Configural invariance	33.579	16	.976	.068 (.035–.100)	.053				
Full metric invariance	34.351	20	.980	.055 (.020–.085)	.051	.772	4	.942	.004
Full scalar invariance	42.764	24	.974	.057 (.027–.085)	.050	8.413	4	.077	.006

Abbreviations: CFI, comparative fit index; RMSEA, root mean squared error of approximation; SRMR, standardized root mean square residual.

* The change in the *χ* ^2^ between models.

^a^ The change in the df (degree of freedom) between models.

^b^ The change in the CFI between models.

**TABLE 6:** Correlations between the studied variables

Variables	1	2	3	4	5	6	7	8	9	10	11	12
(1) ISLES–Footing in the World	1											
(2) ISLES–comprehensibility	0.80^b^	1										
(3) ISLES‐total	0.98^b^	0.90^b^	1									
(4) ISLES‐SF–Footing in the World	0.94^b^	0.73^b^	0.92^b^	1								
(5) ISLES‐SF–comprehensibility	0.77^b^	0.95^b^	0.86^b^	0.69^b^	1							
(6) ISLES‐SF‐total	0.94^b^	0.91^b^	0.97^b^	0.93^b^	0.90^b^	1						
(7) Centrality of Event Scale (CES)	−0.48^b^	−0.40^b^	−0.47^b^	−0.40^b^	−0.44^b^	−0.45^b^	1					
(8) PTSD‐total	−0.66^b^	−0.61^b^	−0.68^b^	−0.61^b^	−0.60^b^	−0.66^b^	0.50^b^	1				
(9) Intrusion	−0.52^b^	−0.52^b^	−0.54^b^	−0.48^b^	−0.53^b^	−0.55^b^	0.44^b^	0.83^b^	1			
(10) Avoidance	−0.13^b^	−0.20^b^	−0.16^b^	−0.09^a^	−0.21^b^	−0.16^b^	0.19^b^	0.44^b^	0.30^b^	1		
(11) Negative alterations in mood and cognitions	−0.65^b^	−0.58^b^	−0.66^b^	−0.61^b^	−0.54^b^	−0.63^b^	0.45^b^	0.91^b^	0.62^b^	0.28^b^	1	
(12) Hyperarousal	−0.60^b^	−0.52^b^	−0.60^b^	−0.54^b^	−0.52^b^	−0.58^b^	0.43^b^	0.87^b^	0.63^b^	0.27^b^	0.70^b^	1

Abbreviation: ISLES, Integration of Stressful Life Experiences Scale.

^a^ Significant at the 0.05 level.

^b^ Significant at the 0.01 level.

### 2.6 Latent mean comparison across gender

Results showed no significant differences between males and females in Footing in the World (CR = .834; *p* = .404; CR = .834; *p* = .404, ISLES and ISLES‐SF, respectively) and comprehensibility (CR = 1.288; *p* = .198; CR = 1.299; *p* = .194, ISLES and ISLES‐SF, respectively).

## 3 DISCUSSION

The current study aimed to translate the ISLES from English into Persian and evaluate the psychometric properties of the 16‐item and 6‐item versions among a sample of university students who have experienced at least one romantic breakup in the past 2 years. The translation process was conducted according to Brislin's (1986) method, and the results confirmed the consistency between the original and the translated versions. The results of qualitative and quantitative face and content validity indicated that the Persian version of the ISLES is relevant and understandable and suitable for assessing meaning‐made after experiencing stressful life events among Iranian university students.

Consistent with our first hypothesis, the CFA results verified that both the 16‐item and 6‐item versions of ISLES consist of two factors: Footing in the World and comprehensibility . These two factors are consistent with Park's (2010) integrative model, which states that the meaning‐making of a life stressor can involve two processes: understanding the meaning of a specific event and finding a sense of significance. The model fit indices proved that the ISLES and ISLES‐SF with two factors fit the data adequately. The factor loadings of all items ranged from 0.48 to 0.87 in ISLES and 0.51 to 0.95 in ISLES‐SF, so all items remained in the measure. The values of Cronbach's alpha and CR showed that the two factors of both ISLES and ISLES‐SF had acceptable internal consistency. In addition, the values of AVE of the two factors of ISLES and ISLES‐SF confirmed acceptable CV for both versions of this scale.

Consistent with our second hypothesis, the extent to which individuals integrate meanings of stressful life events into their global meanings is negatively correlated with PTSD symptoms. This finding is in‐line with previous research (Bellet et al., 2018; Currier et al., 2011; Lancaster & Carlson, 2015) and cognitive theories of PTSD (Dalgleish, 2004) that the inability to integrate traumatic memories into existing cognitive schemas and one's life narrative leads to maladjustment to such experiences. The results of the correlation analyses also revealed that scores on ISLES and ISLES‐SF were negatively correlated with CES, a measure that assesses the degree to which a stressful experience is perceived as a central part of one's identity and life story. This finding is consistent with prior studies (Bellet et al., 2018; Currier et al., 2013; Holland et al., 2010). Previous research has also well documented that CES is strongly positively correlated with posttraumatic symptoms (Berntsen & Rubin, 2007, 2006). On the other hand, meaning‐made is positively associated with mental and physical health (Holland et al., 2010; Holland, Currier, et al., 2014). CES captures the maladaptive accommodation of an event into one's life story, whereas ISLES assesses the adaptive integration of such experiences (Holland et al., 2010).

Consistent with our third hypothesis, the results of measurement invariance analyses demonstrated that both 16‐item and 6‐item versions of ISLES are gender invariant. To our knowledge, Measurement invariance of the ISLES has not been studied in earlier research. The present study established configural, full metric, and full scalar invariances of both 16‐item and 6‐item versions of ISLES across gender groups. This finding demonstrated that the items of ISLES and ISLES‐SF have the same structure and meaning in men and women. The establishment of full scalar invariance within gender groups allowed for comparisons of mean differences. The results of the latent mean comparisons revealed no significant differences in the scores of the two subscales of ISLES and ISLES‐SF between men and women. These findings are consistent with the results reported by Currier et al. (2011), but contrast with those of Holland, Currier et al. (2014), who found that men tended to score significantly higher than women on the comprehensibility factor in both the 16‐item and 6‐item versions of ISLES. In our study, men did have a higher latent mean score on comprehensibility than women, but this difference was not statistically significant. Our study focused exclusively on romantic breakup as a stressful life event, and most research has found no gender differences in experienced distress following breakup between men and women (Simpson, 1987; Sprecher, 1994; Tashiro & Frazier, 2003). Perhaps gender differences in making sense of stressful life events can be found in the context of other stressful events.

This study provides preliminary support for the validity and reliability of both the 16‐item and 6‐item versions of ISLES for assessing adaptive meaning‐made following stressful experiences in Iranian populations. Therefore, the ISLES and ISLES‐SF can be used in research and clinical settings. This measure can also help therapists identify clients who have not adaptively integrated a particular event into their existing cognitive structures and are, therefore, vulnerable to developing negative posttraumatic symptoms.

## 4 LIMITATIONS

The findings should be interpreted taking into account some limitations. First, we relied on a sample of young adults, which limits the generalizability of the findings to other age groups. Future research could examine the factor structure of this measure in older adults, who may exhibit different patterns of the meaning‐made of stressors. Second, this study was limited to a sample that experienced a specific stressor, that is, a romantic breakup. It would be ideal for future research to examine the stability of the factor structure in populations with experiences of other stressful life events. Third, the reliability of the two factors of ISLES and ISLES‐SF was determined by the CR and Cronbach's alpha coefficients. Future research could measure test–retest reliability to further establish the reliability of the two 16‐item and 6‐item versions of ISLES. Fourth, a cross‐sectional design was used in this study. Because meaning‐made is the product of meaning‐making processes that occur over time, future research could use longitudinal designs to capture the dynamic nature of these processes.

## 5 CONCLUSION

This study revealed that both the 16‐item and 6‐item versions of ISLES are efficient instruments with good psychometric properties to assess the outcome of meaning‐making processes after experiencing stressful life events among Iranian university students. The ISLES has the potential to encourage further research on meaning‐making processes and their relationships with a variety of psychological outcomes. Moreover, this measure could help design meaning‐focused interventions to promote the adaptive integration of negative events.

## AUTHOR CONTRIBUTIONS

I confirm that all authors have contributed significantly, and that all authors are in agreement with the content of the manuscript.

## CONFLICT OF INTEREST

The authors declare that there is no conflict of interest.

### PEER REVIEW

The peer review history for this article is available at https://publons.com/publon/10.1002/brb3.2892.

## Data Availability

The data is available at the below link: https://doi.org/10.6084/m9.figshare.21610371.
